# 894. Pain for Burn Patients and Their Pocketbooks: The Financial Toxicity of Outpatient Burn Wound Care

**DOI:** 10.1093/jbcr/irag033.380

**Published:** 2026-04-06

**Authors:** Philip H Chang, Celine Shon

**Affiliations:** William Randolph Hearst Burn Center, NY, New York; Weill Cornell, NY, New York

## Abstract

**Introduction:**

The majority of burn patients in the U.S. are treated as outpatients. Daily dressings changes are still necessary for patients who are ineligible for multiday dressings. Post-COVID pandemic prices significantly increased across the board, and outpatients frequently express concerns about out of pocket expenses including burn dressing supplies which are not always covered by insurance. Our team sought to compare prices of common burn care supplies from both retail pharmacies and online suppliers.

**Methods:**

Two common outpatient burn cases were constructed by our team to evaluate the total cost and pricing variability across five common suppliers (1 online, 4 IRL). The first scenario involved a 4-year-old child with a 3% TBSA scald burn to the chest, requiring daily dressing changes for 14 days. The second scenario involved a 25-year-old adult with a 2% TBSA burn to the left hand dorsal surface, including both the fingers and wrist, requiring dressing change daily for 14 days. The required wound care products were determined to be bacitracin, nonstick dressing, cotton gauze roll, pain medication, and an elastic bandage. For each scenario, standardized quantities and products were analyzed across all five retailers, and a total cost was calculated.

**Results:**

For the first scenario involving the pediatric burn patient, the total cost of a 14 day daily regimen ranged from $122.92 to $229.84. For the second scenario involving adult burn patients, the total cost of a 14 day daily regimen ranged from $156.17 to $267.12. Price variation was greatest with regards to the price of rolled gauze and elastic bandages. In general, retail pharmacies were significantly more expensive compared to online and big-box stores.

**Conclusions:**

Patients find significant differences in price across different retail options with regards to burn dressings, especially with regards to rolled gauze and elastic bandages. While online retail and big box outlets provide lower prices, these options may not always be available because of either geographic constraints or prohibitive costs of online memberships, posing an additional financial hardship for burn patients with limited financial resources.

**Applicability of Research to Practice:**

Price information regarding dressings allows both burn providers and patients to make informed care decisions regarding expected costs of wound care, choices in burn wound management, and future changes in clinical practice to minimize the financial stresses imposed on burn.

**Funding for the study:**

N/A.

